# Physical Activity Using a Wearable Device as an Alternative to Performance Status in Patients With Advanced Lung Cancer

**DOI:** 10.1001/jamaoncol.2024.0023

**Published:** 2024-03-28

**Authors:** Kentaro Ito, Yuta Suzuki, Tadashi Sakaguchi, Kentaro Fujiwara, Yoichi Nishii, Hiroki Yasui, Osamu Taguchi, Osamu Hataji

**Affiliations:** 1 Respiratory Center, Matsusaka Municipal Hospital, Mie, Japan; 2 Department of Biostatistics, Yokohama City University School of Medicine, Yokohama, Japan

## Abstract

**Importance:**

The Eastern Cooperative Oncology Group Performance Status (ECOG PS) is extensively used to guide treatment decisions in patients with advanced lung cancer. However, its assessment is subjective, potentially leading to discordance among observers.

**Objective:**

To investigate the association between measured physical activity and ECOG PS, as well as the potential prognostic value of physical activity measurements in patients with advanced lung cancer.

**Design, Setting, and Participants:**

This single-institution, prospective observational study enrolled 119 patients with advanced lung cancer scheduled to receive systemic therapy as outpatients at Matsusaka Municipal Hospital in Mie, Japan. Participants wore the wearable device amuelink (Sony) for up to 14 days to measure physical activity, including metabolic equivalent tasks, distance walked, and number of steps taken. ECOG PS was assessed at enrollment, which took place from December 2021 to August 2022.

**Main Outcomes And Measures:**

The primary end point was estimating the area under the curve (AUC) for classification into ECOG PS of 2 or higher using physical activity measurements. An analysis of the association with survival was also conducted.

**Results:**

Among the 119 patients (median [range] age, 72 (32-88) years; 71 [59.7%] male), mean distance walked (MDW) had the highest diagnostic value for classifying an ECOG PS of 2 or greater, with an AUC of 0.818 (95% CI, 0.703-0.934). Moreover, MDW was also associated with 6-month survival, with an AUC of 0.806 (95% CI, 0.694-0.918). Survival curves significantly diverged based on the MDW threshold, indicating a potential association with survival outcome (hazard ratio, 0.17; 95% CI, 0.05-0.57).

**Conclusions and Relevance:**

The cohort study suggests that MDW, as measured by a wearable device, was associated with ECOG PS and may serve as a predictor of health status alongside ECOG PS categories. It demonstrates the potential of objectively measured physical activity in complementing subjective ECOG PS assessments in patients with advanced lung cancer. Further research is needed to confirm the prognostic value of physical activity measurements.

## Key Points

**Question:**

Can objectively measured physical activity serve as an alternative to the Eastern Cooperative Oncology Group Performance Status (ECOG PS) for evaluating the general health status of patients with progressive lung cancer, and can these parameters have predictive value for survival outcomes?

**Findings:**

In this observational study of 119 patients and 6 measured physical activity metrics, mean distance walked (MDW) demonstrated the best diagnostic value for classifying an ECOG PS of 2 or higher and was associated with better 6-month survival status. Furthermore, survival analysis based on MDW thresholds showed that physical activity levels could potentially serve as an additional prognostic indicator in oncology.

**Meaning:**

This study suggests that wearable device–measured physical activity, particularly MDW, could potentially supplement the ECOG PS as an evaluative tool for health status in patients with progressive lung cancer and offer predictive insights into survival outcomes.

## Teaser

This cohort study investigates the association between measured physical activity and Eastern Cooperative Oncology Group Performance Status, as well as the potential prognostic value of physical activity measurements in patients with advanced lung cancer.

## Introduction

Deciding the treatment strategy for advanced lung cancer hinges on assessing the general health status in informing therapeutic decision-making. The Eastern Cooperative Oncology Group Performance Status (ECOG PS) is a scoring system classifying general status,^1,2^ which traditionally represents this needed health status. Based on evidence,^3^ some global lung cancer guidelines also refer to the ECOG PS for making treatment decisions.^4,5,6^ Despite its importance, the ECOG PS’s subjective nature often leads to evaluation discrepancies among evaluators. Previous research has explored physical activity’s role in cancer prevention and prognosis improvement,^7,8,9^ but data on its association with survival outcomes remain insufficient. The purpose of this study is to investigate the feasibility of measuring physical activity and the association of ECOG PS or prognosis with physical activity.

## Methods

### Study Design

This is a single-institution, prospective observational study aimed at evaluating the use of physical activity measures for classifying ECOG PS. Enrollment took place from December 2021 to August 2022 at Matsusaka Municipal Hospital in Mie, Japan. Eligible patients were outpatients receiving or about to receive systemic therapy, with exclusion criteria including walking inability due to any reason.

We used amuelink (Sony),^10^ a wearable monitoring device, to measure 6 physical activity metrics: sum and mean of (1) metabolic equivalent tasks, (2) walked distance, and (3) step count. Participants wore the device on their waist from awakening to bedtime for up to 14 days for prospective physical activity observation. Considering patient and family opinions, the investigator/physician objectively scored per the ECOG PS definition.^1^

This study complies with the Declaration of Helsinki principles, ensuring informed consent from all participants. The study is registered with the UMIN Clinical Trial Registry.^11^ For further information, see the eMethods in Supplement 1. We followed the Strengthening the Reporting of Observational Studies in Epidemiology (STROBE) reporting guideline (eTable 1 in Supplement 1).

### Primary End Point

The primary end point was estimating the area under the curve (AUC) for diagnosing ECOG PS of 2 or higher using physical activity in receiver operating characteristic (ROC) analysis, and the secondary end point involved an analysis comparing physical activities across ECOG PS scores. Association of physical activity was also exploratively analyzed. AUC was calculated for each physical activity value to test its association with ECOG PS of 2 or higher and 1 or higher, as well as 6-month survival status. Overall survival was compared between higher- and lower-activity groups, defined by a physical activity threshold calculated with the Youden index.

### Statistical Analysis

Diagnostic value was assessed by AUC in ROC analysis. Survival curves were estimated using the Kaplan-Meier method, and hazard ratios (HRs) were calculated using univariate Cox regression based on dichotomized physical activity values. A sample size was set at 100 patients without a statistical rationale due to a lack of data for calculation. Statistical analyses were performed using SPSS, version 28 (IBM), and tests were conducted 2-sided with statistical significance defined as *P* < .05.

## Results

### Characteristics

A total of 119 patients from 121 enrolled patients were analyzed in this study (eFigure 1 in Supplement 1). The median (range) age was 72 (32-88) years, 71 patients (59.7%) were male, and the majority of patients had an ECOG PS of 0 or 1 (eTable 2 in Supplement 1).

### Association Between Physical Activity and ECOG PS

**Figure 1.**
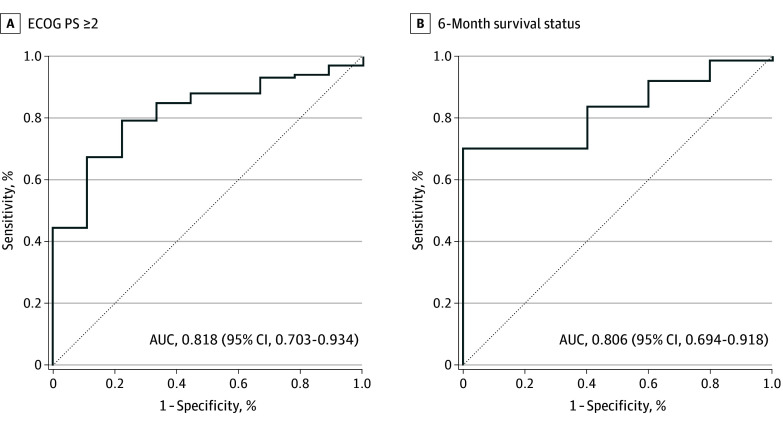
Receiver Operating Characteristic Curves and Area Under the Curve (AUC) Using Mean Distance Walked ECOG PS indicates Eastern Cooperative Oncology Group Performance Status.

AUCs across the measurement of physical activity are summarized in the Table. The mean distance walked (MDW) was the best diagnostic value for an ECOG PS of 2 or more, with an AUC of 0.818 (95% CI, 0.703-0.934; Figure 1A). MDW was also associated with an ECOG PS of 0 or 1 and 6-month survival status, presenting AUCs of 0.765 (95% CI, 0.664-0.863) and 0.806 (95% CI, 0.694-0.918), respectively (Figure 1 and Table). Notable trends were observed in both distance walked and step count across ECOG PS groups (eFigure 2 and eTable 3 in Supplement 1). Consequently, MDW was selected as a possible predictor of ECOG PS and prognosis in this study. The median duration of wearing the device was 8.0 (95% CI, 7.2-8.8) days, with no statistically significant difference in the duration across age or ECOG PS and no change in MDW across the duration (eFigure 3 in Supplement 1).

**Table.** Area Under the Curve in Receiver Operating Characteristic Analysis for Classification of Eastern Cooperative Oncology Group Performance Status (ECOG PS) by Physical Activity

Measurement	Metabolic equivalent tasks		Distance walked		No. of steps	
	Sum (95% CI)	Mean (95% CI)	Sum (95% CI)	Mean (95% CI)	Sum (95% CI)	Mean (95% CI)
ECOG PS 0-1 vs 2-3	0.526 (0.323-0.729)	0.482 (0.337-0.628)	0.784 (0.660-0.908)	0.818 (0.703-0.934)	0.773 (0.627-0.919)	0.755 (0.594-0.915)
ECOG PS 0 vs 1	0.511 (0.398-0.625)	0.593 (0.470-0.716)	0.722 (0.622-0.822)	0.765 (0.664-0.863)	0.763 (0.664-0.863)	0.780 (0.681-0.879)
6-mo Survival	0.712 (0.538-0.887)	0.497 (0.302-0.692)	0.796 (0.660-0.932)	0.806 (0.694-0.918)	0.778 (0.640-0.916)	0.775 (0.640-0.910)

### Physical Activity and Survival Outcomes

**Figure 2.**
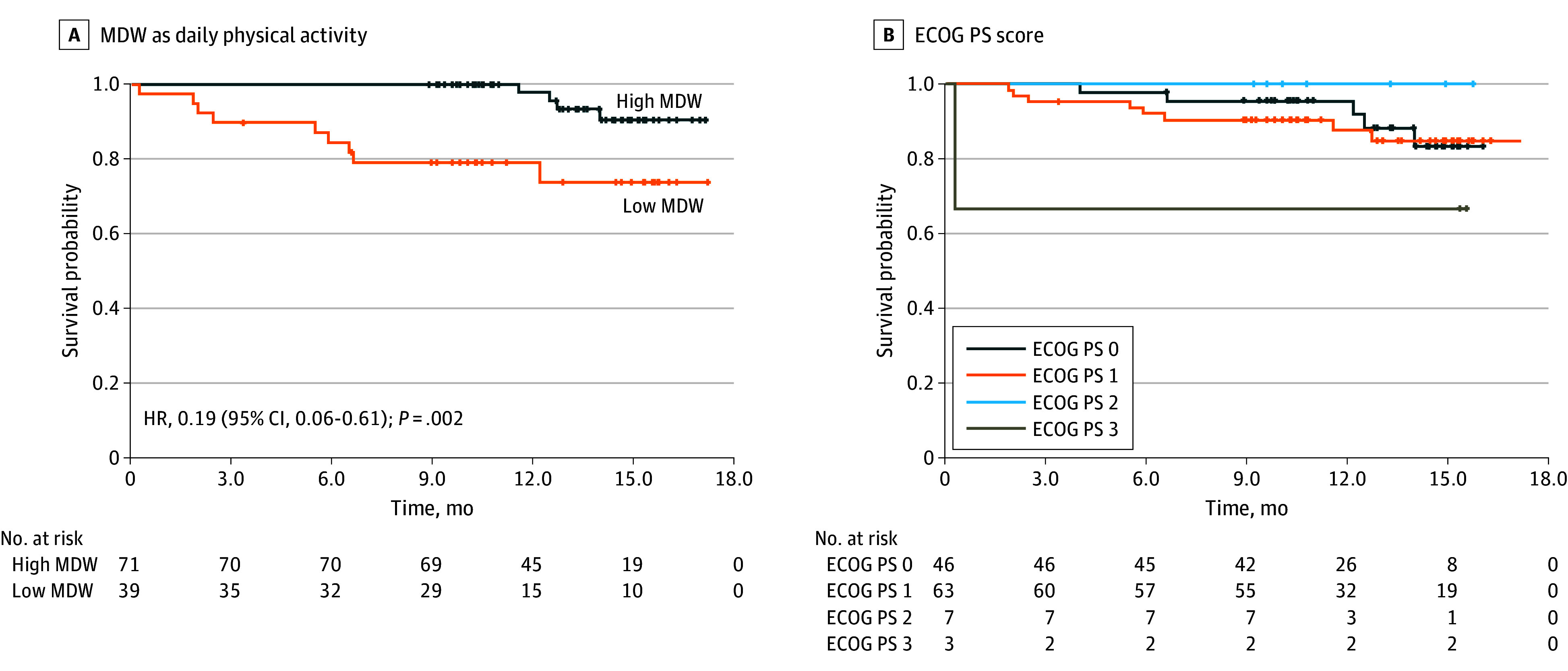
Kaplan-Meier Curves for Overall Survival Overall survival curves based on mean distance walked (MDW) as daily physical activity and Eastern Cooperative Oncology Group Performance Status (ECOG PS). The threshold of MDW was calculated by receiver operating characteristic analysis. HR indicates hazard ratio.

For survival analysis, the median (IQR) follow-up period was 13.4 (10.1-15.2) months, and 14 patients died during this period. Of 119 patients, 1 case was untraceable at 6 months and was treated as a censored case. A statistically significant difference in overall survival was noted between the higher- and lower-activity groups (HR, 0.19; 95% CI, 0.06-0.61; *P* < .002; Figure 2). Conversely, no statistically significant difference in overall survival was observed when comparing subgroups based on other factors including sex, age, smoking status, or ECOG PS (eFigure 4 in Supplement 1). The number of patients with an ECOG PS of 2 or higher was so small that additional subgroup analysis was conducted among patients with an ECOG PS of 0 or 1. The AUC for diagnosing ECOG PS of 0 or 1 and 6-month survival status by MDW were 0.765 (95% CI, 0.670-0.860) and 0.810 (95% CI, 0.700-0.921), respectively (eFigure 5 in Supplement 1). Additionally, a statistically significant difference was observed in overall survival curves between higher- and lower-activity groups (HR, 0.17; 95% CI, 0.05-0.57; *P* < .001; eFigure 6 in Supplement 1). The trend was confirmed both in ECOG PS of 0 and 1 groups (eFigure 7 in Supplement 1).

## Discussion

The present study demonstrated that MDW was associated with ECOG PS and prognosis, indicating its potential as an alternative method for ECOG PS and possible prognostic indicator. Subgroup analysis of patients with an ECOG PS of 0 or 1 also affirmed MDW’s possible prognostic value. Further analysis revealed no difference in survival based on other factors such as age or sex, as depicted in eFigure 4 in Supplement 1. This highlighted the statistically significant contribution of physical activity to survival prediction, irrespective of other factors.

Previous studies have reported an association between step count and prognosis in respiratory diseases,^12,13^ a trend also observed in this analysis. However, MDW appeared to be superior to step count in this study. The wearable device in this study uses a unique algorithm to classify movement types by detecting microvibrations, potentially explaining the unique demonstration of walked distance utility. Additionally, this device does not require other devices or complex operations. Patient understanding, motivation, and geographic location may influence device use and activity levels. However, biases are likely minimized by conducting the study in a single region and due to the aforementioned advantages. Notably, wear duration was not associated with age or ECOG PS (eFigure 3 in Supplement 1).

However, these results do not suggest that physical activity can be an alternative to the ECOG PS scoring system. As seen in eFigure 2 in Supplement 2, a wide range of physical activity among patients even with the same ECOG PS is evident, which indicates that patients with the same ECOG PS would have varying prognoses. We also note the potential for overestimation of the results represented by the *P* value due to the thresholding method. Therefore, we concluded that the measurement of daily physical activity could, however, be used as complementary data for ECOG PS scoring.

### Limitations

This study has several limitations. The follow-up time was insufficient for survival analysis. The treatment line and regimen were various. Few patients with poor ECOG PS were enrolled; further investigation including more patients with ECOG PS of 2 or higher is essential for validation across ECOG PS score.

## Conclusions

This cohort study highlights the potential of objectively measured physical activity as an alternative to ECOG PS and enforces the potential prognostic value of MDW compared with ECOG PS. On observing various levels of physical activity within the same ECOG PS category, we concluded that physical activity could serve as complement data in combination with the ECOG PS score.

## Notes

Supplement 1.
eMethods.
**eTable 1.** STROBE statement **eTable 2.** Characteristics **eTable 3.** Kruskal-Wallis test and multiple comparison test by Dunn-Bonferroni method for each paired score of ECOG PS **eFigure 1.** Flow Chart of the patients **eFigure 2.** Subgroup analyses based on the characteristic factors **eFigure 3.** Wearing duration across ECOG PS and age **eFigure 4.** Subgroup analyses based on the characteristic factors **eFigure 5.** ROC analyses in the patients with ECOG PS of 0 or 1 **eFigure 6.** Survival curves in the subgroup of patients with ECOG PS of 0 or 1 **eFigure 7.** Survival analyses in the patients with ECOG PS of 0 and in the patients with ECOG PS 1

Supplement 2. Data Sharing Statement
