# Supplementary material for: Physical Activity Using a Wearable Device as an Alternative to Performance Status in Patients With Advanced Lung Cancer
Source: JAMA Oncol. 2024 Mar 28;10(5):648–51. doi: 10.1001/jamaoncol.2024.0023 (PMC10979353; doi:10.1001/jamaoncol.2024.0023)
Supplement: Supplement 1. — eMethods. eTable 1. STROBE statement eTable 2. Characteristics eTable 3. Kruskal-Wallis test and multiple comparison test by Dunn-Bonferroni method for each paired score of ECOG PS eFigure 1. Flow Chart of the patients eFigure 2. Subgroup analyses based on the characteristic factors eFigure 3. Wearing duration across ECOG PS and age eFigure 4. Subgroup analyses based on the characteristic factors eFigure 5. ROC analyses in the patients with ECOG PS of 0 or 1 eFigure 6. Survival curves in the subgroup of patients with ECOG PS of 0 or 1 eFigure 7. Survival analyses in the patients with ECOG PS of 0 and in the patients with ECOG PS 1 [file jamaoncol-e240023-s001.pdf]

Supplemental Online Content

Ito K, Suzuki Y, Sakaguchi T, et al. Physical activity using a wearable device as an alternative to performance status in patients with advanced lung cancer. *JAMA Oncol*. Published online March 28, 2024. doi:10.1001/jamaoncol.2024.0023

eMethods.

eTable 1. STROBE statement

eTable 2. Characteristics

eTable 3. Kruskal-Wallis test and multiple comparison test by Dunn-Bonferroni method for each paired score of ECOG PS

eFigure 1. Flow Chart of the patients

eFigure 2. Subgroup analyses based on the characteristic factors

eFigure 3. Wearing duration across ECOG PS and age

eFigure 4. Subgroup analyses based on the characteristic factors

eFigure 5. ROC analyses in the patients with ECOG PS of 0 or 1

eFigure 6. Survival curves in the subgroup of patients with ECOG PS of 0 or 1

eFigure 7. Survival analyses in the patients with ECOG PS of 0 and in the patients with ECOG PS 1

This supplemental material has been provided by the authors to give readers additional information about their work.

© 2024 Ito K et al. JAMA Oncol.

eMethods

Study design

This study is a prospective observational study enrolled patients diagnosed with advanced lung cancer as outpatients for treatment at a single institution between Dec 2021 and Aug 2022. The analysis of this report is about the lung cancer cohort of the prospective observational study to investigate physical activity using a wearable monitoring device in respiratory disease patients, lung cancer, interstitial pneumonia, and COPD. It was planned that the three cohorts be analyzed separately with each having different primary endpoints according to the protocol, and the association between ECOG PS and physical activity was investigated in the lung cancer cohort. The study is registered with UMIN. (UMIN000047834).

Patients.

Eligible criteria is outpatients who were either receiving or about to receive systemic therapy for advanced lung cancer, with exclusion criteria including walking inability due to any reasons. To minimize selection bias, we attempted to enroll consecutive cases whenever possible. Concerned about the occurrence of cases in which physical activity could not be measured correctly, we enrolled as many cases as possible beyond the target number, as long as they were within the enrollment period (Dec 2021-Aug 2022). All enrolled patients provided written informed consent before monitoring physical activity, and they were followed prospectively to evaluate survival outcomes in this study. There were no age-related exclusion criteria for participation in this study. However, individuals who were unable to provide informed consent were excluded, including those who lacked the capacity to understand the study information and those who declined to use the device due to its cumbersome nature.

ECOG PS scoring.

ECOG PS were assessed by the investigator/attending physician at enrollment, taking into account opinions from patients and families, who were provided with the definition of ECOG PS, as below.

The description of ECOG PS Score

Score	Description
0	Fully active, able to carry on all pre-disease performance without restriction.

© 2024 Ito K et al. JAMA Oncol.

1	Restricted in physically strenuous activity but ambulatory and able to carry out work of a light or sedentary nature, e.g., light housework, office work.
2	Ambulatory and capable of all selfcare but unable to carry out any work activities. Up and about more than 50% of waking hours.
3	Capable of only limited selfcare, confined to bed or chair more than 50% of waking hours.
4	Completely disabled. Cannot carry on any selfcare. Totally confined to bed or chair.

* 5. death was omitted.

The PS scores were recorded on the consent form and the electronic medical records, and had not been changed thereafter.

Measures of physical activity using wearable monitoring device.

We used the wearable monitoring device, amuelink (SONY, Tokyo Japan), which can measure a total of 6 physical activity values as follows: sum and mean of (1) METS, (2) distance walked (in meters, m), and (3) number of steps walked (in steps). All participants were instructed to wear the wearable monitoring device for up to 14 days.

Sum was defined as the total amount of physical activity accumulated during the wear period, with calculation of "0" for days when the patient did not wear the device, and "average" is the value of the sum divided by the wear days.

Patients wore the device around their waist, which is recommended by the manufacturer as an appropriate location for assessing step counts. The device was worn from the time they woke up in the morning until they went to bed. At enrollment, the investigator asked them to live a normal life without forced exercise to avoid bias in the estimation of physical activity.

Outcomes.

The purpose of this study is to evaluate the utility of physical activity measures for the classification of ECOG PS, with the primary endpoint of estimating the AUC for classification into ECOG PS of 2 or more using physical activity in the ROC analysis, and the secondary endpoint of correlative analysis comparing the physical activities across ECOG PS scores. In advanced lung cancer, many clinical trials target those with an ECOG of 0 or 1, and this is also reflected in guidelines. Therefore, the classification was primarily focused on ECOG PS ≤1 and PS≥2.

© 2024 Ito K et al. JAMA Oncol.

For selecting the candidate parameter, ROC analysis used the candidate value of physical activity for predicting ECOG PS of 2 or more, and alive state at 6 months. Comparing the AUC, graph, or p-value, the candidate for best prognostic predictor was selected among the measurements of physical activity. Using the selected candidate predictor from the physical activity measurements, we determined threshold values and conducted survival analysis.
After enrollment, we found that few patients with ECOG PS of 2 or more, we added the ROC analysis estimating AUC for diagnosis with ECOG PS of 0. In estimating AUC, the 6-month overall survival status was evaluated in the total monitored patients at the 6-month after enrollment, excluding censored cases. ROC analysis was conducted with SPSS ver.28 (IBM, Inc), which calculates the confidence interval for the AUC using a non-parametric test in SPSS program.
The correlation of physical activity with ECOG PS were assessed with comparison of each physical activity across ECOG PS using Kruskal-Wallis test and multiple comparison test by Dunn-Bonferroni method.
The predictive value of physical activity for survival was examined as an exploratory endpoint, comparing the survival curves based on the threshold of physical activity, and other factors, such as sex, age, smoking status, or ECOG PS. For comparing survival curves, physical activity was binarized, and the two groups were compared. The optimal threshold of physical activity was determined based on Youden's index in the ROC analysis for 6-month survival status. The patients were divided into the higher-activity group and the lower-activity group based on the threshold.

Statistics.
Diagnostic value was assessed to estimate AUC by ROC analysis. Statistical comparisons of continuous values were tested using Kruskal-Wallis test and multiple comparison test by Dunn-Bonferroni method, with p value < 0.05 as a significant difference. Survival curves were estimated using the Kaplan-Meier method, and hazard ratios were estimated using the univariate Cox regression model. In the Cox regression analysis, physical activity was used for binarization before calculating the hazard ratios. Sample size was not calculated based on the statistical rationale, due to the limited information on physical activity investigation. Our study planned to enroll 100 patients as a sample size. Statistical analyses were performed using SPSS ver.28 (IBM, Inc)

Ethics.
The study was conducted in accordance with the principles of the Declaration of Helsinki, and written informed consent was obtained from all enrolled patients. The protocol was

© 2024 Ito K et al. JAMA Oncol.

approved by Institutional Review Board in Matsusaka Municipal Hospital. The study is registered with UMIN. (UMIN000047834).

eTable 1 STROBE statement

	Item No	Recommendation	Page No
**Title and abstract**	1	(*a*) Indicate the study's design with a commonly used term in the title or the abstract	1
		(*b*) Provide in the abstract an informative and balanced summary of what was done and what was found	6-8
**Introduction**			
Background/rationale	2	Explain the scientific background and rationale for the investigation being reported	10
Objectives	3	State specific objectives, including any prespecified hypotheses	10
**Methods**			
Study design	4	Present key elements of study design early in the paper	11
Setting	5	Describe the setting, locations, and relevant dates, including periods of recruitment, exposure, follow-up, and data collection	11
Participants	6	(*a*) Give the eligibility criteria, and the sources and methods of selection of participants. Describe methods of follow-up	11
		(*b*) For matched studies, give matching criteria and number of exposed and unexposed	11
Variables	7	Clearly define all outcomes, exposures, predictors, potential confounders, and effect modifiers. Give diagnostic criteria, if applicable	11
Data sources/ measurement	8*	For each variable of interest, give sources of data and details of methods of assessment (measurement). Describe comparability of assessment methods if there is more than one group	11

© 2024 Ito K et al. JAMA Oncol.

Bias	9	Describe any efforts to address potential sources of bias	15-16
Study size	10	Explain how the study size was arrived at	12
Quantitative variables	11	Explain how quantitative variables were handled in the analyses. If applicable, describe which groupings were chosen and why	12
Statistical methods	12	(*a*) Describe all statistical methods, including those used to control for confounding	12
		(*b*) Describe any methods used to examine subgroups and interactions	12
		(*c*) Explain how missing data were addressed	12
		(*d*) If applicable, explain how loss to follow-up was addressed	eMethod
		(*e*) Describe any sensitivity analyses	eMethod
**Results**			
Participants	13*	(a) Report numbers of individuals at each stage of study—eg numbers potentially eligible, examined for eligibility, confirmed eligible, included in the study, completing follow-up, and analysed	Suppelent
		(b) Give reasons for non-participation at each stage	Supplement
		(c) Consider use of a flow diagram	Supplement
Descriptive data	14*	(a) Give characteristics of study participants (eg demographic, clinical, social) and information on exposures and potential confounders	Supplement
		(b) Indicate number of participants with missing data for each variable of interest	14
		(c) Summarise follow-up time (eg, average and total amount)	14
Outcome data	15*	Report numbers of outcome events or summary measures over time	14

© 2024 Ito K et al. JAMA Oncol.

eTable 2 Characteristics

N	119
Age, median[range]	72 [32-88]
Sex	
Male / Female	71 / 48
Histology	
NSCLC / SCLC	118 / 1
Smoking status	
Current or Former / Never	79 / 40
ECOG PS	
0 / 1 / 2 / 3	46 / 63 / 7 / 3
Treatment line	
CRT	16
0 / 1 / 2/ 3-	2/ 56 / 16 / 28
Other(BSC etc)	1

© 2024 Ito K et al. JAMA Oncol.

eTable 3 Kruskal-Wallis test and multiple comparison test by Dunn-Bonferroni method for each paired score of ECOG PS

Parameter of physical activity to ECOG PS score for null hypothesis	Kruskal-Wallis test	Multiple comparison test (Paired ECOG PS)					
		3 vs. 2	3 vs. 1	3 vs. 0	2 vs. 1	2 vs. 0	1 vs. 0
Sum of METS	0.992	—					
Mean of METS	0.418	—					
Sum of walk distance	0.023	0.771	0.234	0.026	0.119	< 0.001	<0.001
Mean of walk distance	< 0.001	0.694	0.172	0.010	0.095	< 0.001	<0.001
Sum of the walk step	0.001	0.584	0.197	0.013	0.222	< 0.001	<0.001
Mean of walk step	< 0.001	0.442	0.175	0.008	0.368	0.001	<0.001

© 2024 Ito K et al. JAMA Oncol.

eFigure 1 Flow Chart of the patients.

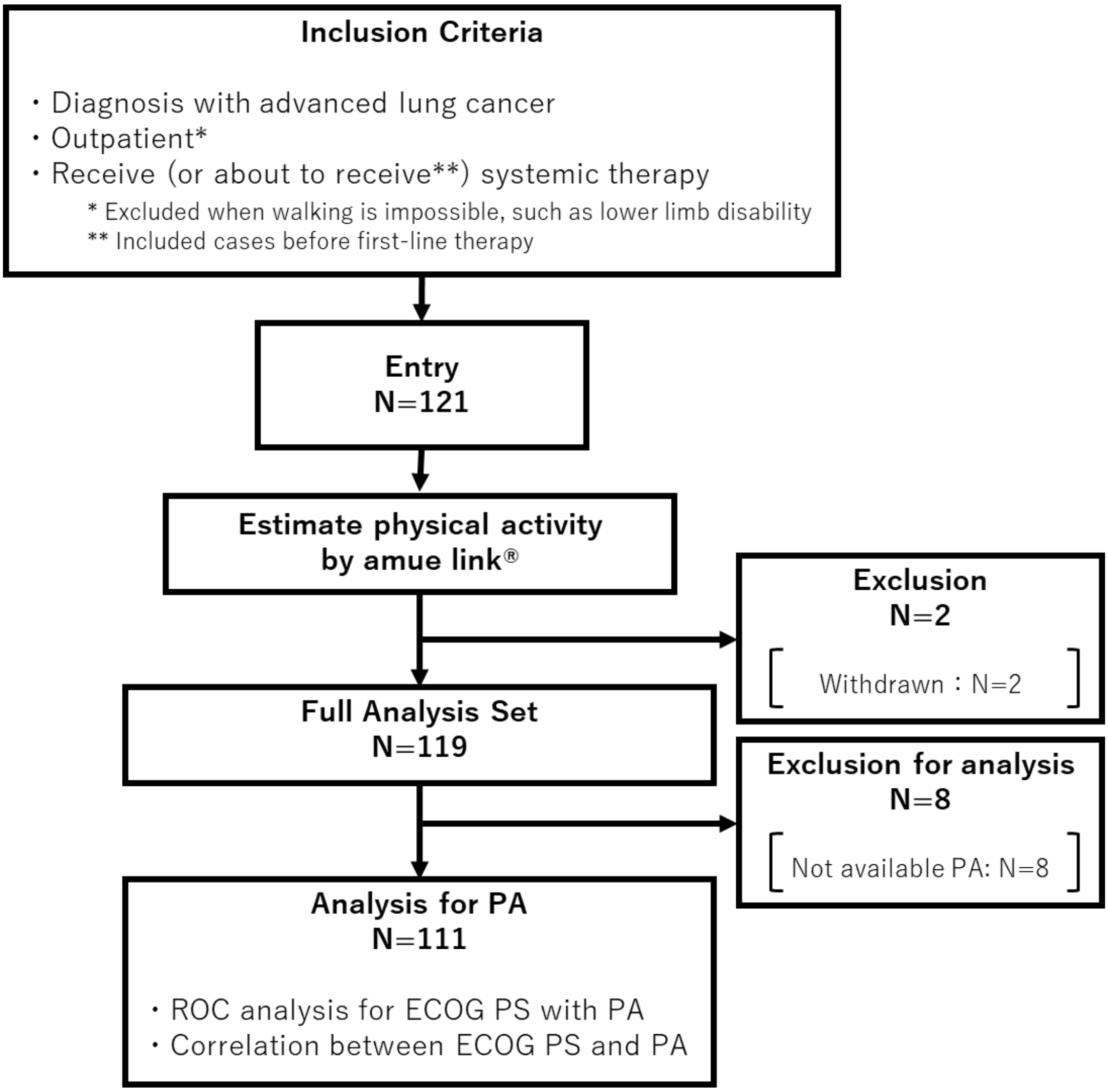

© 2024 Ito K et al. JAMA Oncol.

eFigure 2 Subgroup analyses based on the characteristic factors.

Boxplot of physical activity across ECOG PS score.

METS (left) , walked distance (middle), and walk steps(right) across ECOG PS score were described as sum (in upper) or mean value (in lower), respectively.

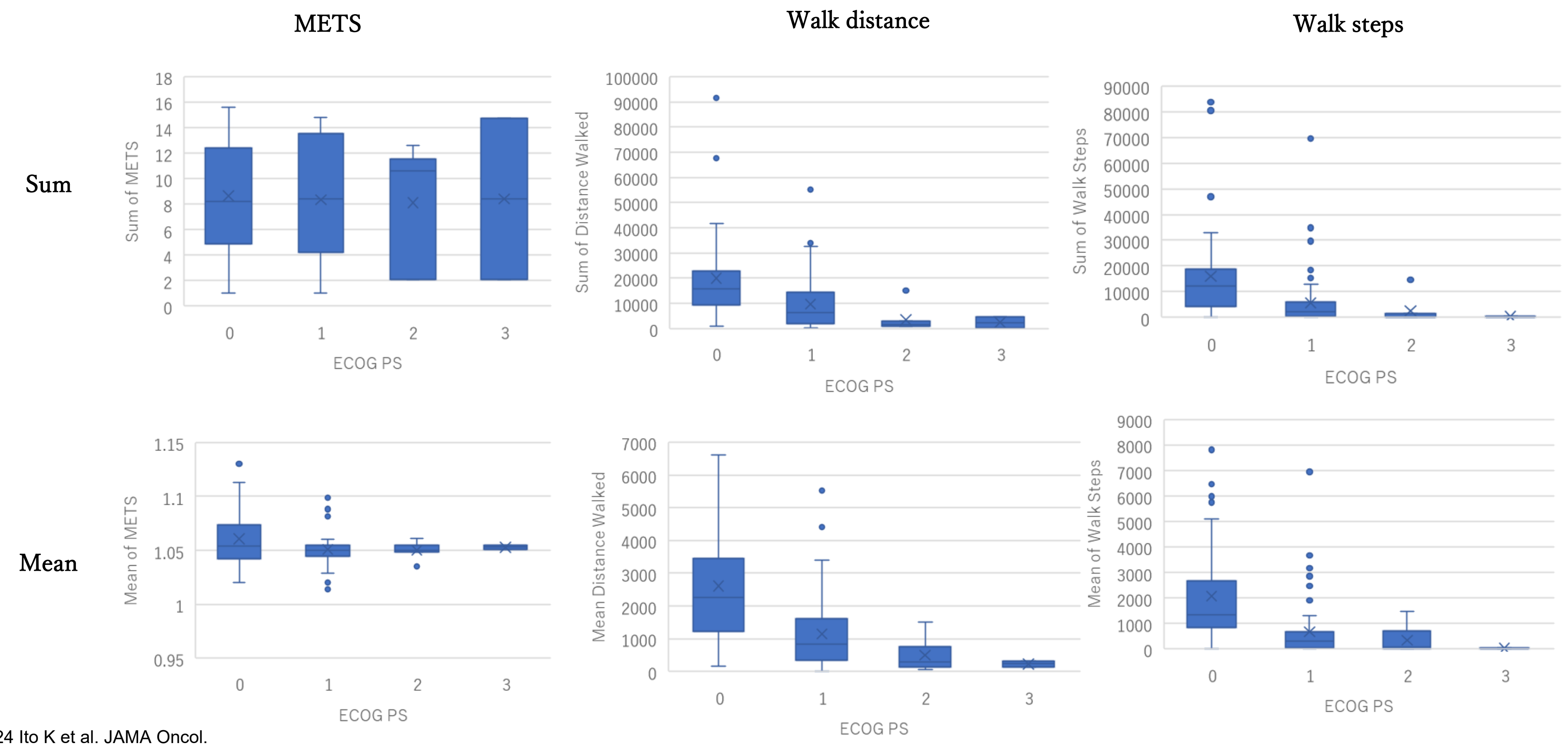

© 2024 Ito K et al. JAMA Oncol.

eFigure 3 **Wearing duration across ECOG PS and age.**

The wearing duration was compared between (A) PS≦1 and PS≧2, (B) PS 0 and PS≧1 and (C) age<75 and age≧75. The comparison of MDW was compared based on wearing duration (D). P value was estimated by t-test.

(A)

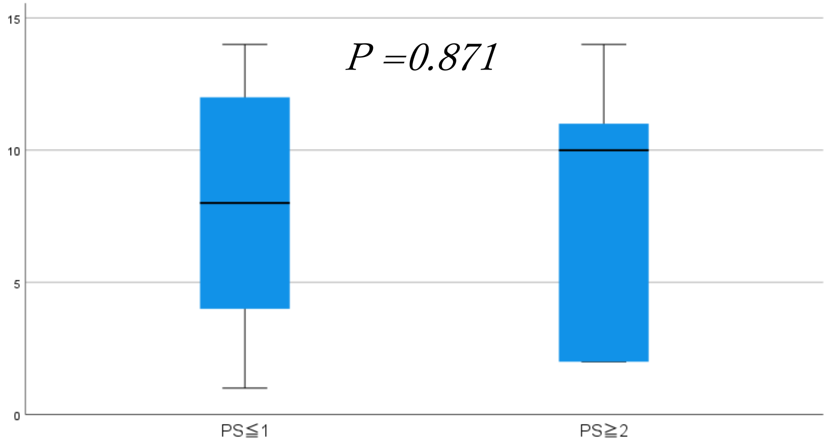

(B)

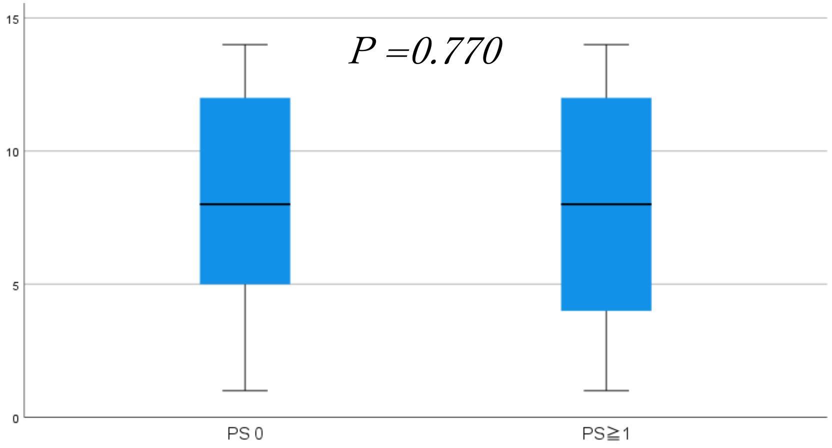

© 2024 Ito K et al. JAMA Oncol.

(C)

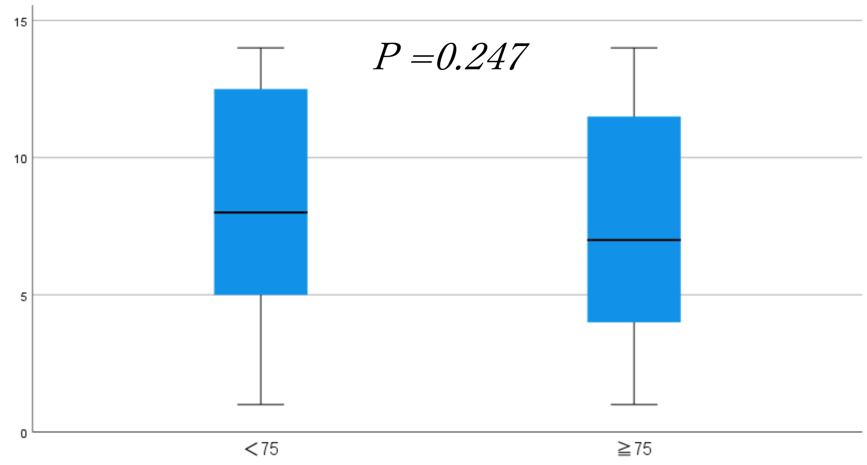

(D)

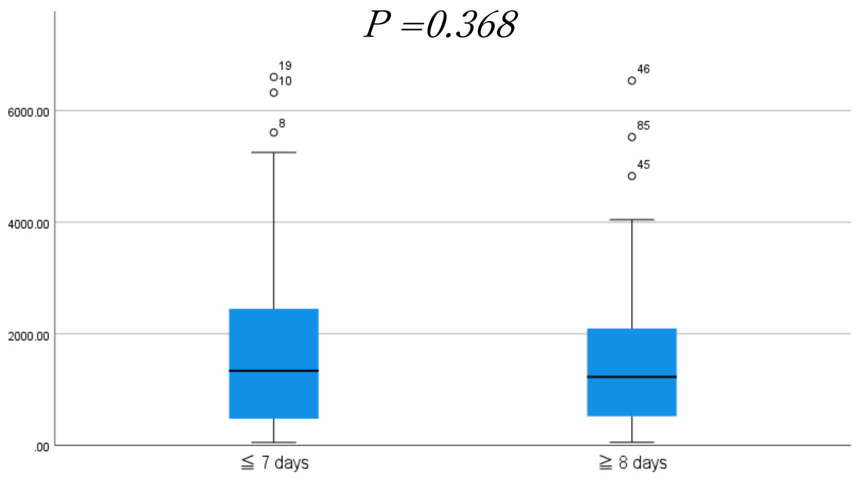

© 2024 Ito K et al. JAMA Oncol.

eFigure 4 **Subgroup analyses based on the characteristic factors.**

Comparing survival curves between (A) Age of ≥75 vs. < 75, (B) Male vs. Female, (C) Smoker vs. Never smoker, and (D) ECOG PS 0 vs. ≥1.

(A)

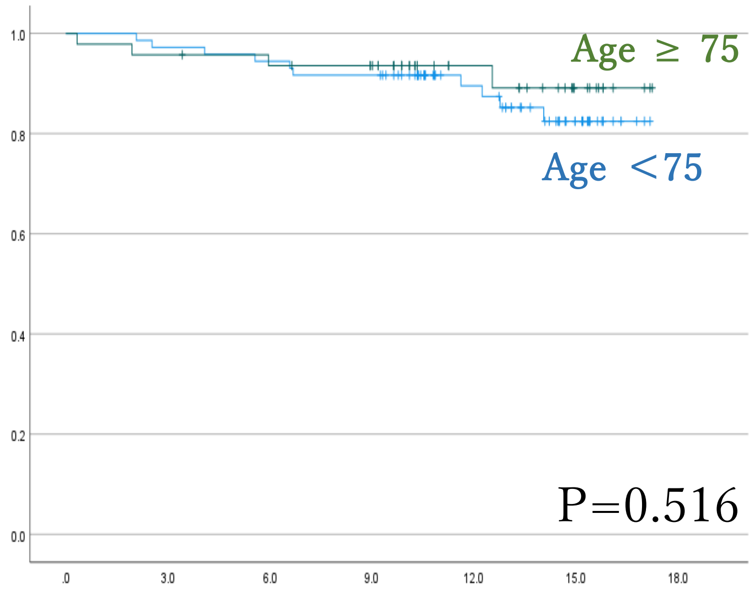

(B)

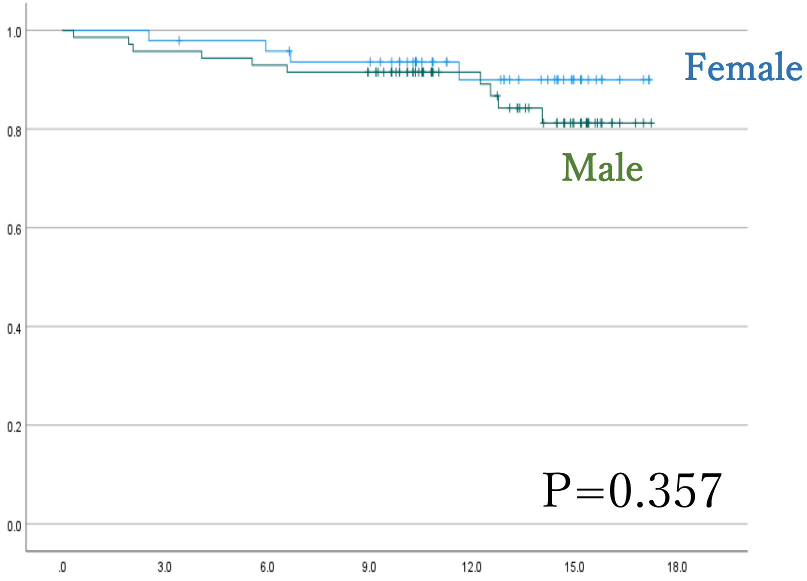

© 2024 Ito K et al. JAMA Oncol.

(C)

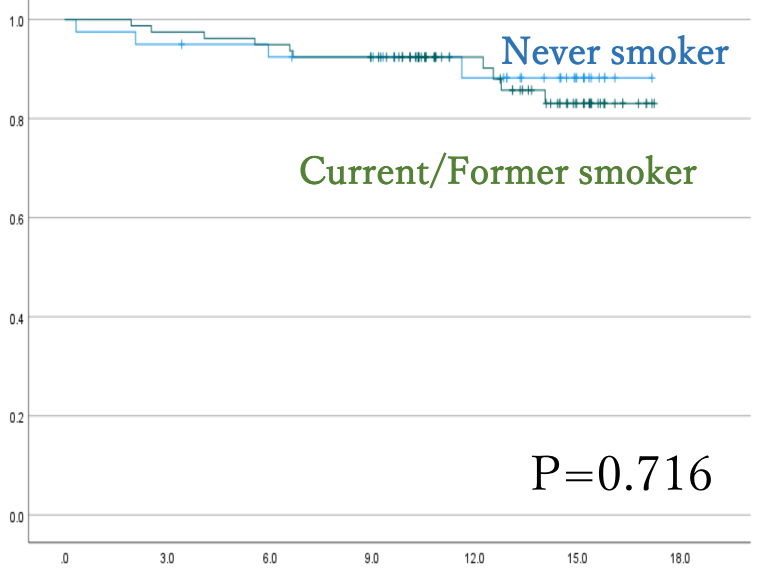

(D

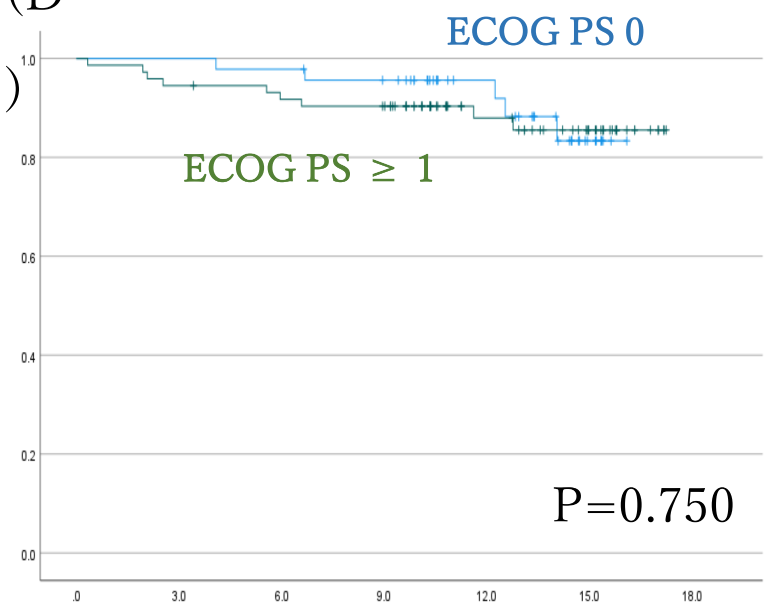

© 2024 Ito K et al. JAMA Oncol.

eFigure 5 ROC analyses in the patients with ECOG PS of 0 or 1. (A) ROC analysis for diagnosis of EOCG PS 0 or 1 by MDW (B) ROC analysis for 6-month survival status by MDW. Survival curves (C) Based on MDW as daily phusical activity , and(D) Based on ECOG PS score.

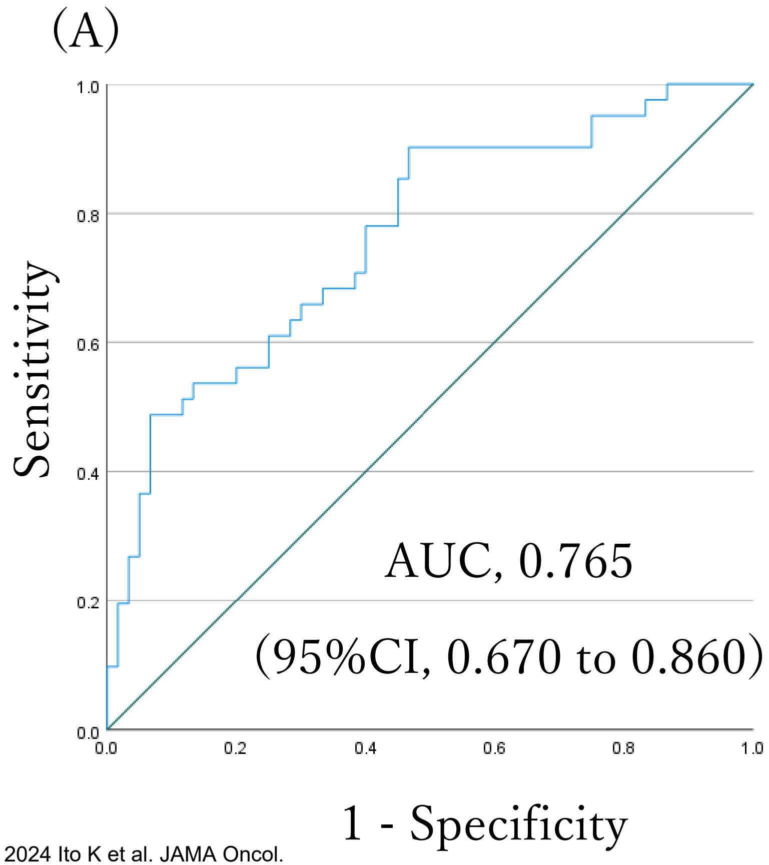

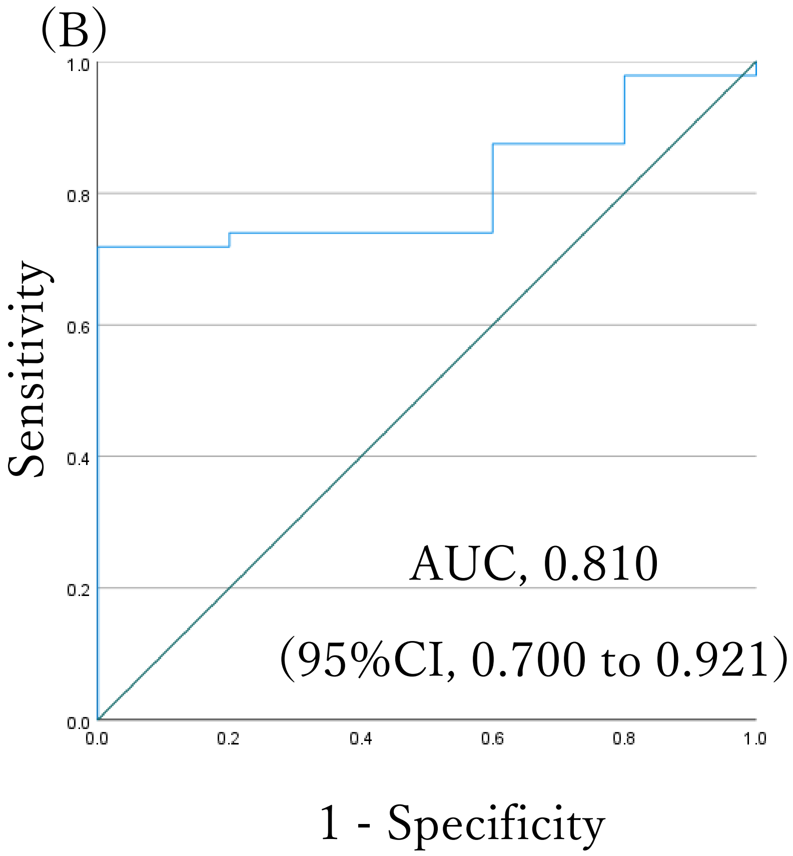

© 2024 Ito K et al. JAMA Oncol.

eFigure 6. Survival curves in the subgroup of patients with ECOG PS of 0 or 1. Survival curves in subgroup of the patients with ECOG PS of 0 or 1, (A) based on MDW and (B) based on ECOG PS score.

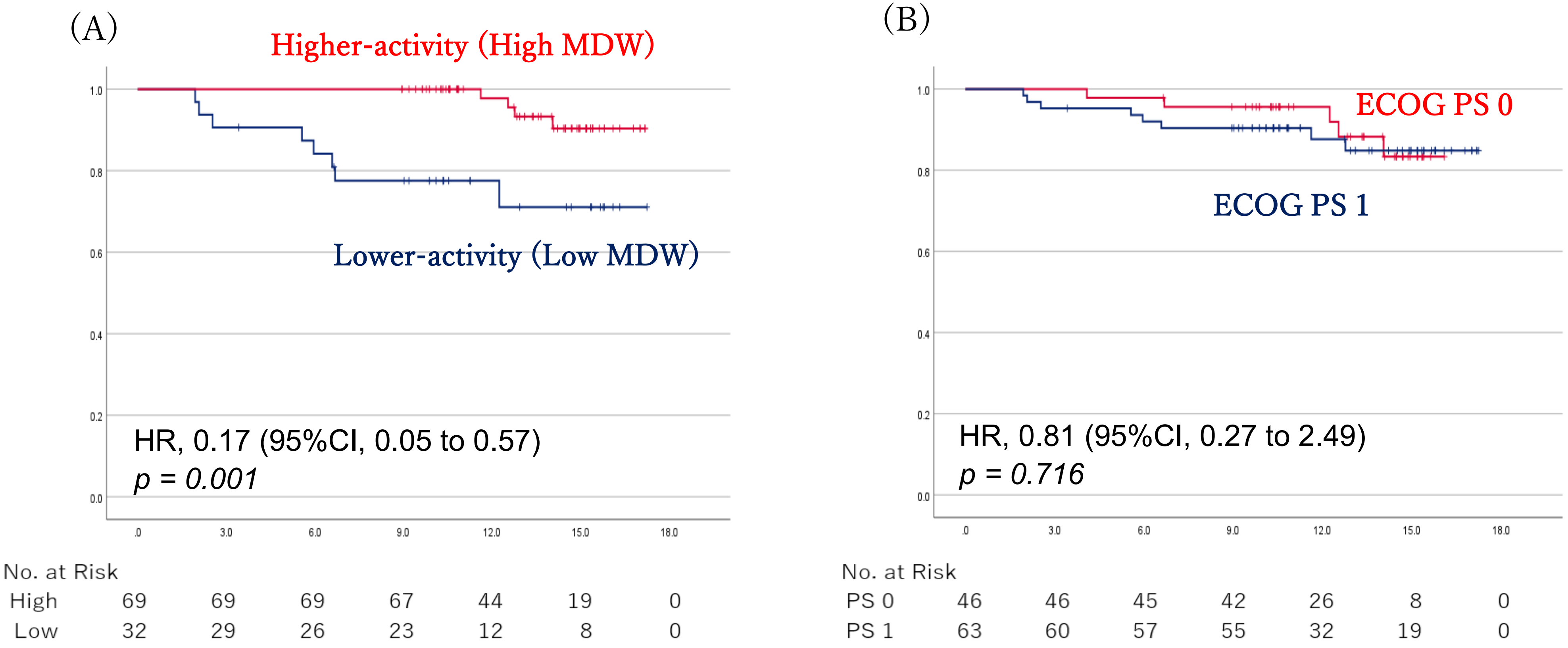

© 2024 Ito K et al. JAMA Oncol.

eFigure 7. Survival analyses in the patients with ECOG PS of 0 and in the patients with ECOG PS 1. (A) Overall survival curves of the patients with ECOG PS of (A) 0 and (B) 1, with being classified by MDW. High and Low were defined by the same threshold in total population.

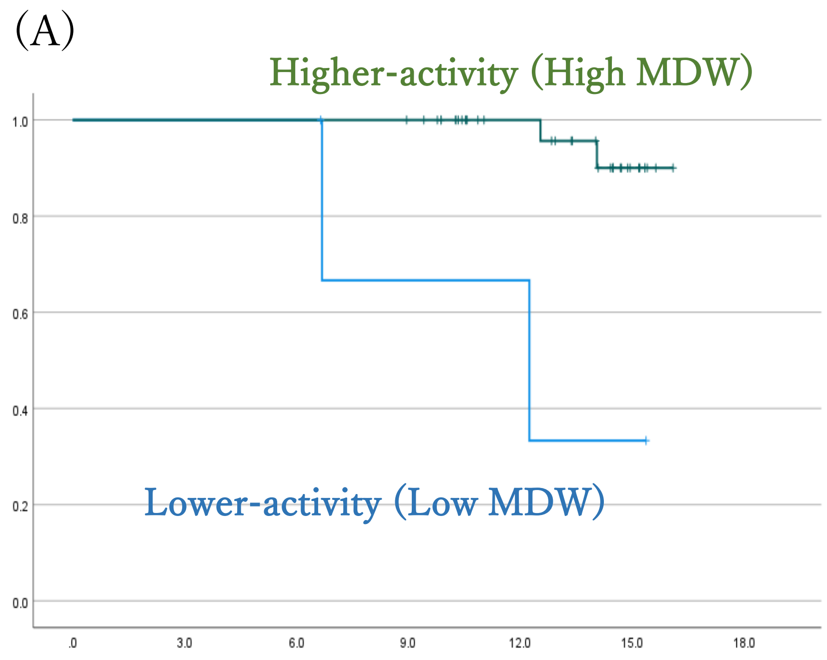

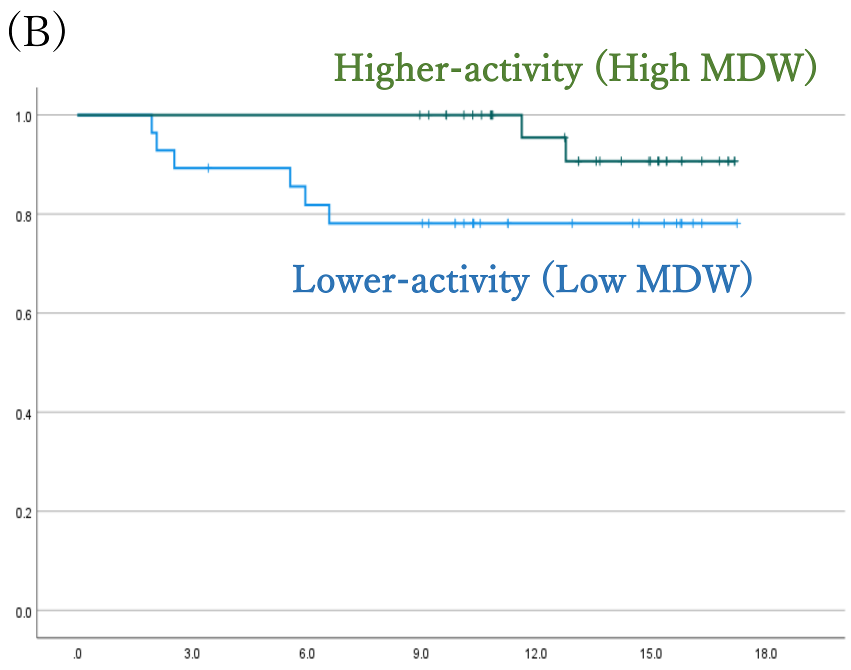

© 2024 Ito K et al. JAMA Oncol.
