# Supplementary material for: Physical Activity Using a Wearable Device as an Alternative to Performance Status in Patients With Advanced Lung Cancer
Source: JAMA Oncol. 2024 Mar 28;10(5):648–51. doi: 10.1001/jamaoncol.2024.0023 (PMC10979353; doi:10.1001/jamaoncol.2024.0023)
Supplement: Supplement 2. — Data Sharing Statement [file jamaoncol-e240023-s002.pdf]

Data Sharing Statement

Ito. Physical Activity Using a Wearable Device as an Alternative to Performance Status in Patients With Advanced Lung Cancer. *JAMA Oncol*. Published March 28, 2024. doi:10.1001/jamaoncol.2024.0023

Data
Data available: Yes
Data types: Deidentified participant data
How to access data: The data will be provided in accordance with the laws on the protection of personal data. The procedure for obtaining the data can be requested by email to the corresponding author E-mail: kentarou_i_0214@yahoo.co.jp
When available: With publication

Supporting Documents
Document types: None

Additional Information
Who can access the data: to anyone requesting the data
Types of analyses: for any purpose
Mechanisms of data availability: with investigator support
Any additional restrictions: Data transfer and analysis will be carried out in accordance with Japan's Personal Information Protection Law.
